# Searching the second hit in patients with inherited retinal dystrophies and monoallelic variants in *ABCA4*, *USH2A* and *CEP290* by whole-gene targeted sequencing

**DOI:** 10.1038/s41598-018-31511-5

**Published:** 2018-09-06

**Authors:** María González-del Pozo, Marta Martín-Sánchez, Nereida Bravo-Gil, Cristina Méndez-Vidal, Ángel Chimenea, Enrique Rodríguez-de la Rúa, Salud Borrego, Guillermo Antiñolo

**Affiliations:** 1 Department of Maternofetal Medicine, Genetics and Reproduction, Institute of Biomedicine of Seville, University Hospital Virgen del Rocío/CSIC/University of Seville, Seville, Spain; 2 Centro de Investigación Biomédica en Red de Enfermedades Raras (CIBERER), Seville, Spain; 3 Department of Ophthalmology, University Hospital Virgen Macarena, Seville, Spain; 4 Retics Patologia Ocular. OFTARED. Instituto de Salud Carlos III, Madrid, Spain

## Abstract

Inherited Retinal Dystrophies are clinically and genetically heterogeneous disorders affecting the photoreceptors. Although NGS has shown to be helpful for the molecular diagnosis of these conditions, some cases remain unsolved. Among these, several individuals harboured monoallelic variants in a recessive gene, suggesting that a comprehensive screening could improve the overall diagnosis. In order to assess the contribution of non-coding variations in a cohort of 29 patients, 25 of them with monoallelic mutations, we performed targeted NGS. The design comprised the entire genomic sequence of three genes (*USH2A*, *ABCA4* and *CEP290)*, the coding exons of 76 genes and two disease-associated intronic regions in *OFD1* and *PRPF31*. As a result, likely causative mutations (8 novel) were identified in 17 probands (diagnostic rate: 58.62%), including two copy-number variations in *USH2A* (one deletion of exons 22–55 and one duplication of exons 46–47). Possibly damaging deep-intronic mutations were identified in one family, and another with a monoallelic variant harboured causal mutations in a different *locus*. In conclusion, due to the high prevalence of carriers of IRD mutations and the results obtained here, sequencing entire genes do not seem to be the approach of choice for detecting the second hit in IRD patients with monoallelic variants.

## Introduction

Inherited Retinal Dystrophies (IRDs) are a group of rare disorders characterized by the progressive loss of photoreceptors in the retina, with a prevalence of 1 in 3,000 individuals worldwide^1^. Depending on the first photoreceptor cell affected, IRDs are subdivided in rod-cone and cone-rod degenerations. The most common form of IRDs is Retinitis Pigmentosa (RP), a rod-cone disease defined by a primary death of rods, which results in night blindness and constriction of the visual field. Later in life, loss of cones leads to a decreased visual acuity^2^. RP can be inherited as an autosomal dominant (adRP), autosomal recessive (arRP) or X-linked (xlRP) trait, but in a large percentage of cases the mode of inheritance is unknown due to absence of family history (simplex RP, sRP). In other pathologies like cone-rod dystrophies (COD), cones degenerate first, whereas in Leber congenital amaurosis (LCA) both types of photoreceptors are damaged simultaneously^3^. Maculopathies like Stargardt disease (STGD) are defined by loss of central vision and accumulation of yellow flecks deposits around the macula^4,5^. There are also syndromic pathologies related to IRDs such as Usher syndrome (USH), in which RP is accompanied by congenital hearing impairment^2^. Several of these conditions share some features, which leads to overlapping phenotypes. Moreover, IRDs are characterized by huge phenotypic variability, in which clinical features, age of onset and disease progression can vary from patient to patient, even in the same family (inter- and intra-familial variability)^6,7^. Furthermore, IRDs are one of the most genetically heterogeneous disorders. To date, more than 300 genes have been associated (RetNet; https://sph.uth.edu/retnet/, accessed July 2018). In addition, mutations in a single gene can be associated with a broad phenotypic spectrum and a specific phenotype can be caused by mutations in multiple genes^8^.

Next-generation sequencing (NGS) resulted in an improvement of the diagnostic rate of this group of heterogeneous disorders^9–11^. However, even though these strategies show high efficacy in a large proportion of cases, around 40–50% of cases remain unsolved^9,12^. Deep-intronic variants, large rearrangements that escape genetic detection or currently unknown IRDs genes may explain these cases^13^. In order to increase the diagnostic rate of this group of disorders, other genomic regions not routinely analyzed must be considered.

Aberrant splicing is a well-known disease-causing mechanism. In fact, it is estimated that a significant percentage of mutations related to monogenic pathologies have an effect on splicing^14,15^. Indeed, deep-intronic mutations in *ABCA4*^16,17^, *CEP290*^18^, *USH2A*^19^, *CHM*^20,21^, *PRPF31*^22^ or *OFD1*^23^ have been described as disease-causing in IRDs. Furthermore, mutations in genes coding for spliceosome components have been found in patients with RP^24,25^, which enlightens the importance of alternative splicing in this condition. Structural variants, including CNVs, have also been described as a relevant cause of disease^26^. Specifically in IRDs, duplications and deletions have been linked to the development of syndromic and non-syndromic cases^20,27–29^. These mutations are easier to detect by whole-genome or whole gene sequencing, as coding and non-coding elements analysis allows the identification of the accurate size and both breakpoints of CNVs.

Here we applied a targeted gene panel covering the entire genomic region of three genes *(ABCA4*, *USH2A* and *CEP290*) and the coding exons of 76 additional genes for the molecular analysis of 29 IRDs patients with simplex or suspected autosomal recessive inheritance. For 25 of them, other methods have previously succeeded in identifying a heterozygous variant in one of these genes. Our study aimed to find a second variant in the same gene to explain the patients’ phenotype by compound heterozygous inheritance. The diagnostic rate was 58.62%% (17/29), of which 14 cases (~77.7%) were solved by the identification of *USH2A* mutations.

## Results

### Clinical features

All analyzed families (n = 29) were of Spanish origin. Index patients received a well-defined clinical diagnosis, which included either RP, USH type II (USHII), STGD, LCA or COD. Available clinical findings of the index patients of the likely genetically diagnosed families are reported in Table 1. The presumed underlying mode of inheritance was either autosomal recessive or simplex in all families. In 10 cases, DNA samples from additional family members were used for segregation analysis of the candidate variants.

**Table 1:** Clinical and genetic findingsin the index patients of the likely characterized families.

Family (index)	Onset age: First symptom Extraction age: Symptoms	Fundus examination	Clin. Diagn.	Gene	[Allele 1]	[Allele 2]	Clin. Significance (Known v.)*	Segr An.	Other features and comments
					Reference	Reference	Pathog. Scores (Novel v.)**		
A (II:1)	10y: VAD. 34y: VA CF; NB; VFR; CVA;	Macular pigment deposits	STGD	*ABCA4*	M1: c.4253 + 5G > A; r.(spl?)^68^	M2: c.5898G > A; r.(spl?) p.Glu1966Glu **This study**	**M1** Clinvar: Pathog.	Yes	Photoph.
							**M2** MT: Damaging NNS: Donor Lost HSF: Site Broken		
B (II:1)	2y: Intense photoph. 5y: VAD; CVA.	No apparent changes	COD	*CNGB3*	M3: c.1148del; p.Thr383Ilefs*13^53^		**M3** Clinvar: CIP [Pathog.(14); VUS(1)]	Yes	Consang.; Micronystagmus; Amblyopia; Hypermetropia
				*ABCA4*	m4: c.466A > G; p.Ile156Val (†)^44^		**m4** Clinvar: VUS	—	
C (II:1)	40y: NB 69y: VFC.; VAD.	Typical of RP at a later stage; RPE atrophy	sRP	*FSCN2*	m5: c.1345 + 6_1345 + 10dup; r.(spl?); **This study**		**m5** MT: Damaging NNS: No changes HSF: No impact	NA	Catar.
				*ABCA4*	m6: c.6148G > C; p.Val2050Leu (†)^69^		**m6** Clinvar: CIP [Ben.(1); Likely ben.(4); Likely pathog.(3); Pathog.(1)]	NA	
D (II:1)	1y: NB 29y: Ring scotoma; VAD; CVA.	Punctate yellow-white deposits in the macula; Peripapillary atrophy	LCA	*LRAT*	M7: c.163C > G; p.Arg55Gly **This study**		**M7** MT: Damaging SIFT: Damaging Polyph: Damaging	Yes	Photoph.; Consang.
E (II:4)	18y: NB 24y: Tunnel vision (central island, 30°)	RPE atrophy, bone spicule pigmentation	sRP	*USH2A*	M8: c.1560C > A; p.Cys520* **This study**	M9: c.2276G > T; p.Cys759Phe (†)^62^	**M8** MT: Damaging	NA	None
							**M9** Clinvar: CIP [Likely pathog.(6); Pathog.(7); VUS(2)]		
				*USH2A*	m10: c.6590 C > T; p.Thr2197Ile^20^		**m10** Clinvar: CIP [Likely Pathog.(1); VUS(1)]	NA	
F (II:3)	25y: NB 39y: VFC; Discrete VAD.	Bone spicule pigmentation in the periphery	sRP	*USH2A*	**M11**: c.2167 + 5G > A; r.(spl?)^70^	M9: c.2276G > T; p.Cys759Phe (†)^62^	**M11** Clinvar: Pathog^71^	NA	Myopia; Astigmatism
							**M9**: see above		
G (II:1)	18y: VF constr. 48y: NB; Tunnel vision; VAD	Typical of RP	sRP	*USH2A*	M9: c.2276G > T; p.Cys759Phe (†)^62^	M12: c.12574 C > T; p.Arg4192Cys^72^	**M9**: see above	NA	Incipient catar.; Tritanopia
							**M12** Clinvar: CIP [Likely pathog.(2); VUS(1)		
H (II:3)	12y: NB 33y: VFC.; VAD; CVA	Bone spicule pigmentation in the periphery	arRP + SNHL	*USH2A*	M9: c.2276G > T; p.Cys759Phe (†)^62^	M13: c.12457del; p.Ala4153Profs*14	**M9**: see above	Yes	Progressive and bilateral SNHL (33y); Father with SNHL. Brother with isolated arRP
							**M13** HGMD: Pathog.		
I (II:2)	30y: VF constr. 50y: NB; VF island 5° central; VAD	Typical of RP	sRP	*USH2A*	M9: c.2276G > T; p.Cys759Phe (†)^62^	M14: c.9799T > C; p.Cys3267Arg^73^	**M9**: see above	NA	Catar.; Photoph.
							**M14** Clinvar: Likely pathog.		
J (II:1)	19y: NB 30y: VFC.	Typical of RP	sRP	*USH2A*	M9: c.2276G > T; p.Cys759Phe(†)^62^	M15: c.11156G > A; p.Arg3719His^72^	**M9**: see above	NA	None
							**M15** Clinvar: Pathog.		
K (II:1)	12y: NB and VFR. 15y: VF Central island, 10°	Decrease in retinal thickness; No bone-spicule pigmentation	sRP (sp)	*USH2A*	M9: c.2276G > T; p.Cys759Phe (†)^62^	M16: c.14011G > T; p.Glu4671* This study	**M9**: see above	Yes	None
							**M16** MT: Damaging		
L (II:1)	43y: NB 53y: VFC; VAD.	Typical of RP	sRP	*USH2A*	M9: c.2276G > T; p.Cys759Phe (†)^62^	M14: c.9799T > C; p.Cys3267Arg^73^	**M9**: see above	Yes	Catar.
							**M14**: see above		
M (II:1)	14y: NB 28y: VFC.; VAD.	Typical of RP	USH	*USH2A*	M17: c.2299del; p.Glu767Serfs*21 (†)^74^	M18: c.15089C > A; p.Ser5030*^75^	**M17** Clinvar: Pathog./Likely pathog.	Yes	Nystagmus; Bilateral SNHL
							**M18** LOVD: Pathog.		
N (II:12)	39y: VFC. 49y: NB	Typical of RP	arRP	*USH2A*	M17: c.2299del; p.Glu767Serfs*21 (†)^74^	M19: c.4325T >C; p.Phe1442Ser^76^	**M17**: see above	NA	Diabetes mellitus (Type II)
							**M19** LOVD: Likely Pathog.		
O (II:1)	7y: NB 58y: Tunnel vision (5°); VAD.; CVA	Bone spicule pigmentation and pallor of the optic disc	sRP	*USH2A*	M20: c.907C > A; p.Arg303Ser^61^	M9: c.2276G > T; p.Cys759Phe (†)^62^	**M20** LOVD: Likely Pathog.	NA	Photoph.;Catar.; Aphakia; Glaucoma
							**M9**: see above		
P (II:9)	13y: NB 35y: Tunnel vision (central island, 10°); VAD.	Typical of RP	USH	*USH2A*	M20: c.907C > A; p.Arg303Ser (†)^61^	M21: Duplication Ex 46–47c.9055 + 100_9371 + 5544dup; p.? **This study**	**M20**: see above	Yes	Strabismus; Astigmatism; Photoph.;Catar.; Two sisters with isolated arRP
							**M21** MT: Damaging		
Q (II:4)	33y: NB 34y: VFC	Typical of RP at a later stage	arRP	*USH2A*	M20: c.907C > A; p.Arg303Ser (†)^61^	M22: c.12067–2A > G; r.spl^77^	**M20**: see above	Yes	Incipient catar. Shiny ILM
							**M22** Clinvar: Pathog.		
R (II:6)	10y: NB 62y: VFC; LP; Legal Blindness	Typical of RP	USH	*USH2A*	M23: Deletion Ex 22–55c.4628-2287_10939 + 3867del; p.? **This study**		**M23** MT: Damaging	Yes	Catar.; Nystagmus; Bilateral SNHL
S (II:1)	14y: NB 30y: VDA, VFR	Typical of RP	sRP	*USH2A*	m33: c.5363A > G; p.Asp1788Gly^30^	m39: c.6806-810A > G; r.? m40: c.6050-8058G > C; r.?	**m33** MT: Benign SIFT: Benign Polyph: Possibly Damaging	No	Myopia, astigmatism
							**m39** MT: Benign NNS: New donor HSF: New Donor		
							**m40** MT: Benign NNS: New acceptor HSF: New acceptor		

^*^The clinical significance of the known variants identified has been obtained using Clinvar, LOVD or HGMD databases.

**In order to predict the impact on the protein’s function of the novel variants, we have conducted *in silico* analysis using MutationTaster (MT) for all kind of mutations, SIFT and Polyphen (Polyph) for the missense variant, and NNSPLICE (NNS) and HSF for the splice site variants.

Alt: Alteration; arRP: Autosomal recessive Retinitis Pigmentosa; Catar.: cataracts; CF: Counting fingers; CIP: Conflicting interpretations of pathogenicity; Clin: Clinical; COD: Cone Dystrophy; CVA: Colour vision Alteration; D: Damaging or Disease causing; DL: Donor lost; ERM: Epiretinal membrane; LCA: Leber Congenital Amaurosis; LP: Light perception; ILM: Internal limiting membrane; MT: MutationTaster; N: Neutral; NA: Not available; NB: Night Blindness; NR: No response; Pathog.: Pathogenicity; Photoph.: Photophobia; Polyph: Polyphen; RP: Retinitis Pigmentosa; RPE: Retinal pigment epithelium; SB: Site broken; Segr. An.: Segregation Analysis; SNHL: Sensorineural hearing loss; sp: *sine pigmento*; sRP: Simplex RP; STGD: Stargardt disease; USH: Usher Syndrome; v: variant; VA: Visual acuity; VAD: Visual Acuity Decresed; VFC: Visual Field Contriction; VFR: Visual Field Reduction; VUS: Variant of unknown significance; y: Years;(w.a.): when available; (†): Variant previously detected by other techniques.Uppercase “M#” indicates likely causal mutations, lowercase “m#” indicates other variants. Fundus typical of RP comprised: Bone spicule pigmentation, narrowed vessels and pallor of the optic disc.

### NGS data quality

**Figure 1:**
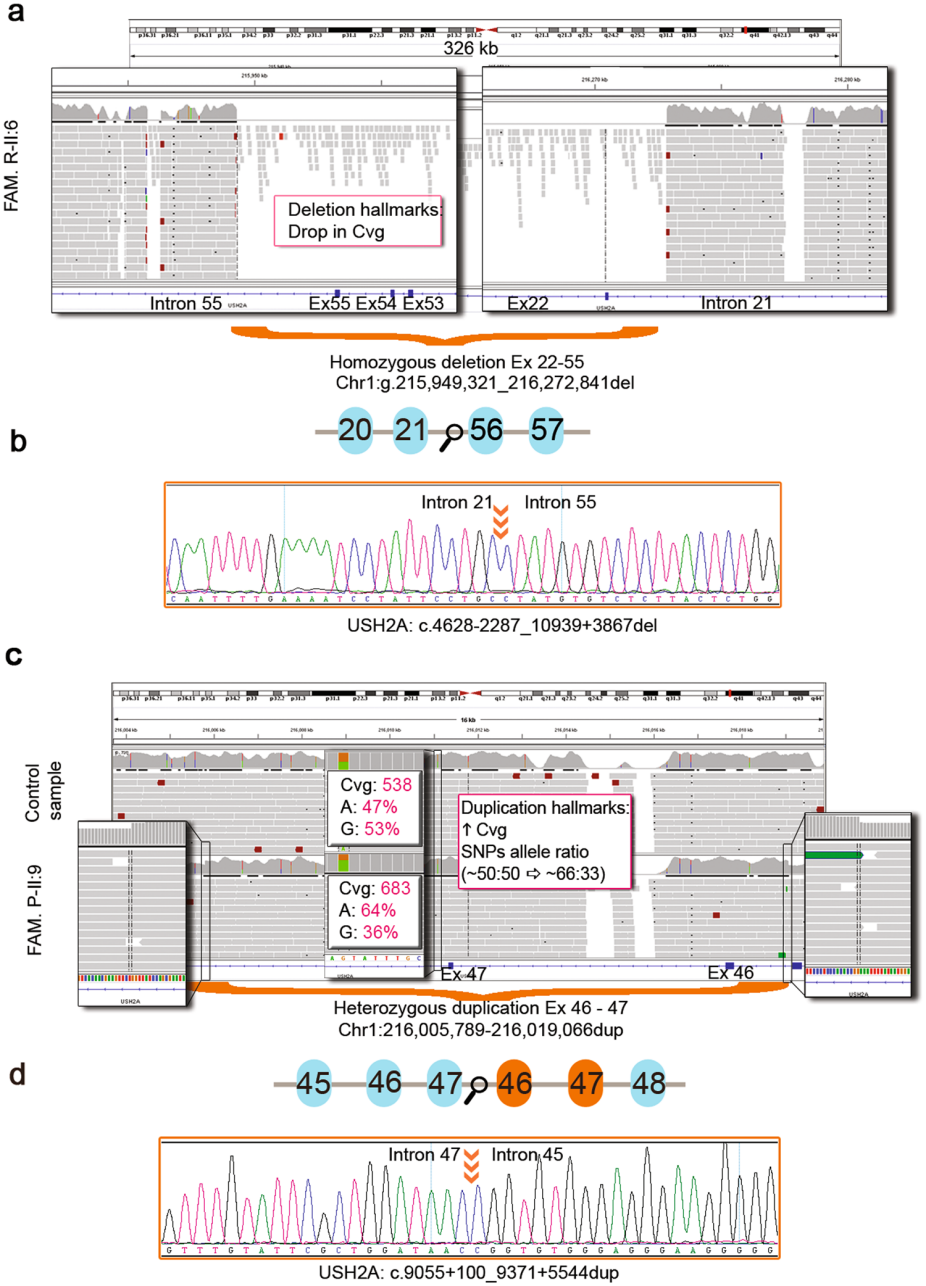
Detection of CNVs in *USH2A* using our NGS approach. (**a**) IGV snapshot showing the homozygous deletion of exons 22–55 detected in family R-II:6 (Chr1:g.215,949,321_216,272,841del, hg19). The capture of whole genomic sequence of *USH2A* allowed us to determinate the CNVs breakpoints. (**b**) Schematic representation of the mutated gDNA sequence and Sanger sequencing of the breakpoint area (orange arrows) confirming the *USH2A* deletion (c.4628-2287_10939 + 3867del; NM_206933). (**c**) IGV snapshot showing the heterozygous duplication of exons 46–47 (Chr1:g.216,005,789_216,019,066dup, hg19) detected in family P-II:9 versus a control sample. The heterozygous duplication can be inspected visually using IGV paying special attention to (i) a sharp increase in the coverage and (ii) changes in the allele ratios of all the SNPs within the duplicated interval from ~50:50 to ~67:33 unmasking the presence of a total of three copies. (**d**) Schematic representation of the mutated gDNA sequence and Sanger sequencing of the breakpoint area (orange arrows) confirming the *USH2A* tandem duplication of exons 46 and 47 (c.9055 + 100_9371 + 5544dup; NM_206933).

The panel design covered 95.3% (1,346,725 bp) of 1,412,505 target bases. The uncovered bases represented 4.7% of the total number of bases, most of them lying in non-coding regions (repeating elements: Long terminal repeats, LTRs and Long interspersed nuclear elements, LINEs). The specific uncovered bases of the genes *ABCA4, CEP290* and *USH2A* are provided in Supplementary Table S1. Only one exonic region in exon 15 (ORF15) of *RPGR* remained uncovered (98–145 bp, depending on the patients). The overall mean coverage was 809X with 100% of captured bases covered, except for individual II:6 of family R, whose coverage dropped to 96.6% due to a large homozygous deletion (Fig. 1). NGS performed on an Illumina MiSeq or NextSeq systems achieved on average 9,828,453 reads per run of which 8,213,528 were mapped on target (82.73%).

### Validation of the panel

Twenty-five out of the 29 cases included in this study carried heterozygous mutations previously detected by other techniques (Table 2), and so, they were used as positive controls for our approach. The application of the data analysis pipeline allowed the accurate re-detection of all the known variants, indicating a mutation detection rate of 100%.

**Table 2:** Initial clinical diagnosis and monoallelic variants identified by other approaches in each of the probands included in the study.

Family ID (index)	Clinical diagnosis	Previous variants	Previous studies	Second variant in the same gene	Solved with this panel
A (II:1)	STGD	—	None	—	Yes (*ABCA4*)
B(II:1)	COD	m4:*ABCA4* c.466A > G; p.Ile156Val	Complete sequencing by NGS of coding exons of *ABCA4* and *CRX* (External genetics laboratory - Imegen, S.L.)	No	Yes (*CNGB3*)
C (II:1)	sRP and unilateral maculopathy	m6:*ABCA4* c.6148G > C; p.Val2050Leu	Targeted Sequencing Panel^30^	No	Unknown (*FSCN2*)
D (II:1)	LCA	—	None	—	Yes (*LRAT*)
E (II:4)	sRP	M9:*USH2A* c.2276G > T; p.Cys759Phe	Sanger Sequencing of USH2A exon 13	Yes	Yes
F (II:3)	sRP	M9:*USH2A* c.2276G > T; p.Cys759Phe	Targeted Sequencing Panel^30^ [Bravo-Gil *et al*., 2017]	Yes	Yes
G (II:1)	sRP	M9:*USH2A* c.2276G > T; p.Cys759Phe	Sanger Sequencing of USH2A exon 13	Yes	Yes
H (II:3)	arRP	M9:*USH2A* c.2276G > T; p.Cys759Phe	Sanger Sequencing of USH2A exon 13	Yes	Yes
I (II:2)	sRP	M9:*USH2A* c.2276G > T; p.Cys759Phe	Sanger Sequencing of USH2A exon 13	Yes	Yes
J (II:1)	sRP	M9:*USH2A* c.2276G > T; p.Cys759Phe	Sanger Sequencing of USH2A exon 13	Yes	Yes
K (II:1)	sRP sine pigmento	M9:*USH2A* c.2276G > T; p.Cys759Phe	Sanger Sequencing of USH2A exon 13	Yes	Yes
L (II:1)	sRP	M9:*USH2A* c.2276G > T; p.Cys759Phe	Sanger Sequencing of USH2A exon 13	Yes	Yes
M (II:1)	USHER	M17: *USH2A* c.2299del; p.Glu767Serfs*21	Sanger Sequencing of USH2A exon 13	Yes	Yes
N (II:12)	arRP	M17: *USH2A* c.2299del; p.Glu767Serfs*21	Genotyping microarray for arRP (584 known variants, Asper Biotech, Ltd)	Yes	Yes
O (II:1)	sRP	M9:*USH2A* c.2276G > T; p.Cys759Phe	Sanger Sequencing of USH2A exon 13	Yes	Yes
P (II:9)	USHER	M20:*USH2A* c.907 C > A; p.Arg303Ser	Genotyping microarray for arRP (584 known variants, Asper Biotech, Ltd)	Yes	Yes
Q (II:4)	arRP	M20:*USH2A* c.907 C > A; p.Arg303Ser	Targeted Sequencing with the same panel as^30^ [Bravo-Gil *et al*., 2017]	Yes	Yes
R (II:6)	USHER	—	None	—	Yes (*USH2A*)
S (II:1)	sRP	m33:*USH2A* c.5363A > G; p.Asp1788Gly	Targeted Sequencing Panel^30^ [Bravo-Gil *et al*., 2017]	No	Unknown (*USH2A*)
T (II:1)	sRP	m26:*ABCA4* c.5881G > A; p.Gly1961Arg//m27:*CEP290* c.2691A > G; p.Ile897Met	Targeted Sequencing Panel^30^ [Bravo-Gil *et al*., 2017]	No	No
U (II:3)	sRP	m28:*ABCA4* c.5882G > A p.Gly1961Glu	Targeted Sequencing Panel^30^ [Bravo-Gil *et al*., 2017]	No	No
V (II:1)	sRP	m29:*ABCA4* c.5908 C > T; p.Leu1970Phe	Targeted Sequencing Panel^30^ [Bravo-Gil *et al*., 2017]	No	No
W (II:3)	sRP	m6:*ABCA4* c.6148G > C; p.Val2050Leu	Targeted Sequencing Panel^30^ [Bravo-Gil *et al*., 2017]	No	No
X (II:1)	sRP	m6:*ABCA4* c.6148G > C; p.Val2050Leu	Targeted Sequencing Panel^30^ [Bravo-Gil *et al*., 2017]	No	No
Y (II:1)	arRP	m30:*CEP290* c.3517C > A p.Gln1173Lys	Targeted Sequencing Panel^30^ [Bravo-Gil *et al*., 2017]	No	No
Z (II:1)	sRP	m31:*CEP290* c.4237G > C; p.Asp1413His	Targeted Sequencing Panel^30^ [Bravo-Gil *et al*., 2017]	No	No
AA (II:4)	sRP	m32:*USH2A* c.1486A > G; p.Thr496Ala	Targeted Sequencing Panel^30^ [Bravo-Gil *et al*., 2017]	No	No
AB (II:1)	STGD	—	None	—	No
AC (II:3)	STGD	m4:*ABCA4* c.466A > G; p.Ile156Val	Targeted Sequencing Panel^30^ [Bravo-Gil *et al*., 2016]	No	No

arRP: Autosomal recessive Retinitis Pigmentosa; COD: Cone Dystrophy; LCA: Leber Congenital Amaurosis; sRP: Simplex RP; STGD: Stargardt disease. Uppercase “M#” indicates likely causal mutations, lowercase “m#” indicates other variants.

### Identification and assessment of candidate variants

In order to identify likely disease-causing variants for each sample, we conducted a stepwise mutation detection protocol as previously described^9,30^ with some modifications: heterozygous mutations in genes with one previous detected variant were prioritized and new variants, including CNVs, were looked for in non-coding regions (deep-intronic and splicing mutations) and coding regions (synonymous variants). Intronic variants were analyzed with *in silico* tools for their potential effect on splicing. If no mutations were found, variants in other genes were assessed as we described in the Methods section.

Sequencing of the gene panel led to the identification of a mean of 2,349 potential variants per patient. After filtering out common polymorphisms with MAF > 0.015 in any of the variant databases queried, including 1,000 Genomes, ExAC and EVS, an average of 314 rare variants per sample remained, of which a range from 1 to 4 were prioritized as described above to be co-segregated by Sanger sequencing.

**Figure 2:**
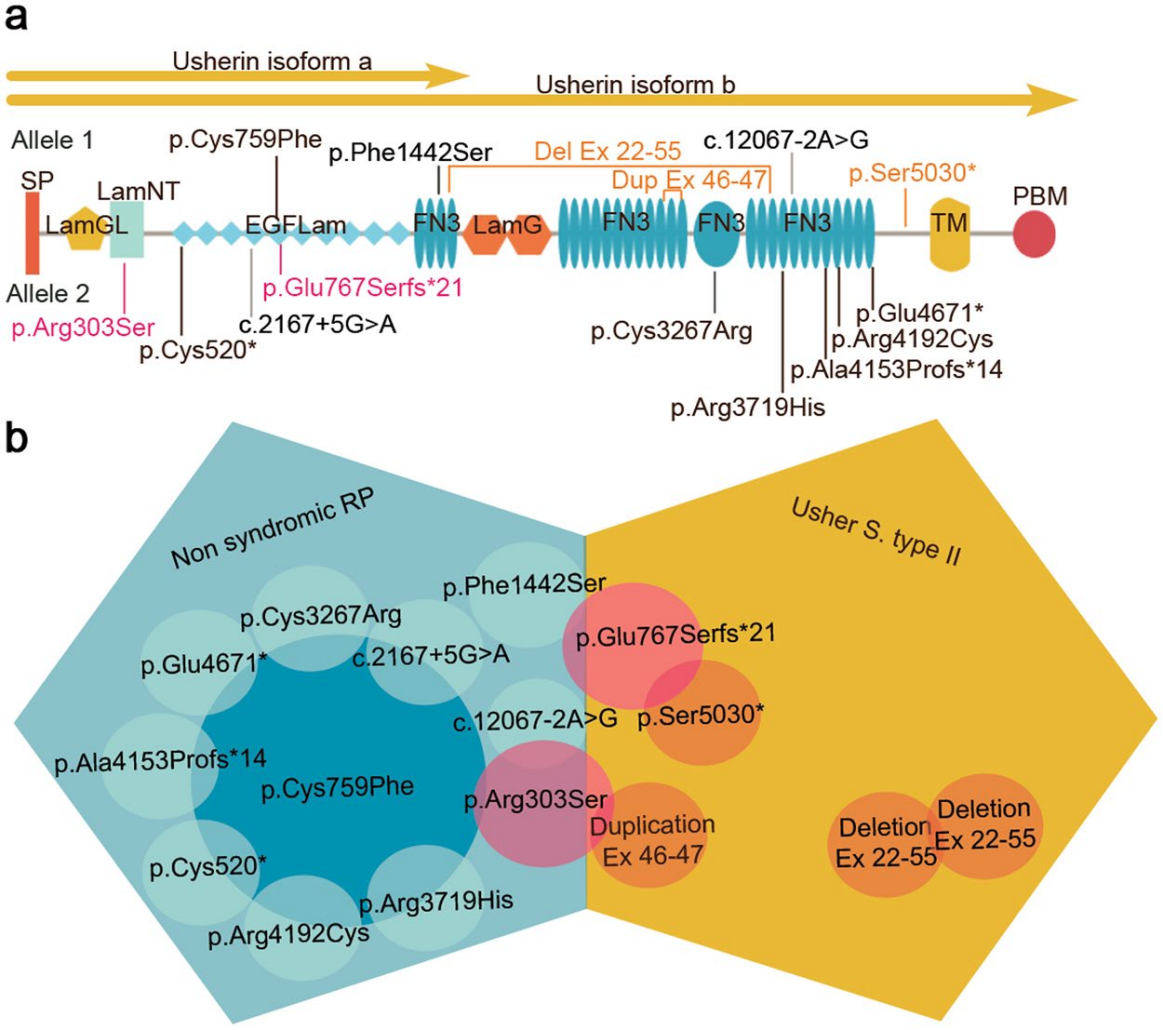
*USH2A* mutations and genotype-phenotype correlations. (**a**) Schematic representation of usherin structure showing the mutated residues located within different protein domains. Isoform “a” is an N-terminal fragment of isoform “b”. Mutations in orange font are implicated in USHII, mutations in fuchsia font are associated with both non-syndromic RP and USHII and mutations in black font are associated with non-syndromic RP. SP: signal peptide; LamGL: LamG-like jellyroll fold; Lam NT: Laminin N-terminal; EGFLam: Laminin-type EGF-like (LE); FN3: fibronectin type-III; LamG: Laminin G; TM: Transmembrane domain; PDB: PDZ-binding domain. (**b**) Phenotype-genotype correlations of usherin mutations. Variants responsible of non-syndromic RP are represented in blue. The variant p.Cys759Phe has been detected in combination with other deleterious alleles in nine patients with non-syndromic RP. Mutations shown in fuchsia color have been detected in individuals with both non-syndromic RP and USH depending on the nature of the second variant. Variants responsible of USHII are shown in orange.

As a result, 31 pathogenic mutations were identified as likely causative in 17 probands (six familial cases and 11 simplex cases) (Supplementary Fig. S1) achieving a diagnostic rate of 58.62% (Table 1). The most frequently mutated gene in this study was *USH2A* (Table 3), and the most prevalent mutation was p.Cys759Phe. We found this mutation in nine patients, always in a compound heterozygous state with a deleterious allele, and only in non-syndromic RP patients (Fig. 2). All novel and known sequence variants of the genetically diagnosed patients have been submitted to the Leiden Open Variation Database, LOVD (https://databases.lovd.nl/).

**Table 3:** Distribution of the likely causative genes in our IRD cohort.

Clinical diagnosis	Solved cases/Total number of cases	Mutated genes (number of cases)
ar Retinitis Pigmentosa	3/4	*USH2A (3)*
simplex Retinitis Pigmentosa	8/17	*USH2A (8)*
Leber congenital amaurosis	1/1	*LRAT (1)*
Stargardt disease	1/3	*ABCA4 (1)*
Usher Syndrome	3/3	*USH2A (3)*
ar Cone dystrophy	1/1	*CNGB3 (1)*

ar: Autosomal recessive.

Among the candidate variants, two of them were CNVs in *USH2A*, comprising one homozygous deletion of exons 22–55 (c.4628–2287_10939 + 3867del) and one heterozygous duplication of exons 46 and 47 (c.9055 + 100_9371 + 5544dup) (Fig. 1). Furthermore, we also detected 18 missense, four frameshift, three nonsense, three intronic-splicing and one synonymous variant located in the exonic canonical splice site (Table 1). Eight of the 31 variants were novel and absent in public databases (ClinVar, LOVD, Pubmed, dbSNP, ExAC, GnomAD, EVS, 1000G and CSVS). Specifically, we detected three homozygous (probands of families B, D and R) and 28 compound heterozygous mutations in autosomal recessive IRDs genes. However, segregation analysis could only be performed in 10 out of 17 families, due to the difficulty in recruiting additional family members in simplex cases. In the remaining patients, candidate variants were presumed to be disease-causing since they correlated with their specific phenotype and they met the established pathogenicity criteria (see Methods section). In this study, we emphasize the importance of intronic and synonymous variants and their effect on splicing processes, which let us diagnosed two cases.

Besides the 31 disease causing mutations, we detected one heterozygous mutation in an autosomal dominant IRDs-associated gene (*FSCN2*, c.1345 + 6_1345 + 10 dup) in one proband affected of simplex RP (Family C-II:1). Nevertheless, segregation analysis could not be performed in this family and the *in silico* predictions failed to validate this mutation as a splice-altering variant (Table 1). Interestingly, this patient was also a carrier of the *ABCA4* allele (p.Val2050Leu) reported as variant with conflicting interpretations of pathogenicity in the Clinvar database. Therefore, additional studies are needed to ascertain the genetic cause of the disease in this family.

### Deep-intronic variants assessment

One of the main objectives of this study was to gain knowledge of the contribution of deep-intronic variants in our population. After analyzing the genomic data, only those deep-intronic variants that fulfilled the selection criteria to be considered causative mutations were selected to be segregated, when possible. A total of 5 variants were found to pass the standards: m33 (*USH2A* c.6326–17446_6326-17439dup) in family B-II:1, m36 (*ABCA4* c.66 + 2044G > A) in family X-II:1, m37 (*CEP290* c.3104-238 T > G) in family Z-II:1 and m39 (*USH2A* c.6806-810 A > G) and m40 (*USH2A* c.6050-8058G > C) in family S-II:1 (Supplementary Fig. S1). Variants m33, m36 and m37 do not segregate with the disease, as they were in *cis* with the previously found mutation in these patients. Regarding the other family (Family S), although segregation analysis could not be conducted, both variants (m39: *USH2A* c.6806-810 A > G and m40: *USH2A* c.6050-8058G > C) are predicted to induce the activation of a cryptic donor/acceptor site respectively. Moreover, a very low frequency in the queried databases was retrieved for these changes (0 and 13 heterozygous carriers in GnomAD, respectively).

### Detection of the second mutation in patients with monoallelic variants

Among the families with a monoallelic variant in one of the genes included in the panel, 15 harboured a single previously detected variant in *USH2A*, eight in *ABCA4* and two in *CEP290* (Table 2). Remarkably, 13 out of 15 of the families with a previous *USH2A* variant were genetically solved by the identification of a second hit in the same gene. Interestingly, family S harbours, besides a missense mutation (c.5363 A > G, p.Asp1788Gly) in *USH2A*, two deep-intronic variants that, although additional studies are needed, might be causative.

However, the diagnostic yield dropped in families with a known variant in *ABCA4* and *CEP290*, since the second potentially causative mutations were not detected in these genes. Of note, one of the cases (Family B) with a known *ABCA4* mutation (c.466 A > G; p.Ile156Val) was solved by the identification of a homozygous likely disease-causing mutation in another gene: *CNGB3* (c.1148del; p.Thr383Ilefs*13) (Table 1).

### Clinical heterogeneity of *USH2A* mutations

In our study, we found the *USH2A* p.Cys759Phe allele in compound heterozygosis in 9 patients with non-syndromic RP (Fig. 2). Three patients who received a USHII diagnosis harboured a combination of one nonsense and one frameshift mutation (family M), a homozygous CNV (family R) and one CNV and a missense mutation (family P) in *USH2A*. Remarkably, not all the affected members of the family P fulfilled the diagnostic criteria of USH, since two affected siblings (II:1 and II:7) exhibited a less severe phenotype consisting of non-syndromic RP (Supplementary Fig. S1). Another family with a significant intrafamilial variability was family H. In this family, only two of the three affected siblings (II:3 and II:4) suffered from RP and hearing loss (Table 1). However, the hearing loss was more likely to have a different genetic cause since the father (individual I:1) of the index patient (II:3) was affected of progressive and bilateral non-syndromic hearing loss. Additionally, here we describe two *USH2A* mutations, p.Glu767Serfs*21 and p.Arg303Ser, found in patients with syndromic and non-syndromic RP.

## Discussion

In this study, we conducted a NGS targeted sequencing approach comprising all exons of 76 retinal disease genes, three entire genes (*USH2A*, *ABCA4* and *CEP290*), and two deep-intronic regions located in *OFD1* and *PRPF31*, to identify the genetic cause of 29 Spanish patients of IRDs, most of them carrying a monoallelic variant in *USH2A*, *ABCA4* and *CEP290*.

The molecular diagnosis was achieved in 58.62% of IRDs patients (17/29). This diagnostic yield is in line with previous works^30^ that similarly analyzed population-specific IRDs genes, and it is somewhat higher than other studies involving more genes^31,32^. It demonstrates that a consistent and adapted design of the panel guarantees a good diagnostic yield while reducing sequencing costs, time and analytical effort.

In our cohort, we detected 31 likely disease-causing mutations. The majority of them were missense variants (58.1%), followed by splicing (12.9%), and frameshift variants (12.9%). Nonsense and CNVs represented 9.6% and 6.5%, respectively. The increasing number of reported deep-intronic mutations in IRD genes prompted us to include three entire genes in our panel design: *ABCA4*, *USH2A* and *CEP290*. These genes accumulate a high number of pathogenic deep-intronic variants reported in the literature^18,19,33,34^. In this group of patients, five deep-intronic variants that met the pathogenicity criteria were detected in four families (Supplementary Fig. S1). However, in three of them (families B, X and Z) the segregation analysis discarded their role in the disease aetiology. The index patient of family S (II:1) was clinically diagnosed of non-syndromic RP and harboured a rare missense variant (c.5363 A > G; p.Asp1788Gly) in *USH2A* previously detected by targeted NGS (Table 2). Regarding the missense mutation, only two heterozygous individuals have been identified in GnomAD, and had no entry neither in Clinvar nor LOVD. Sequencing the *USH2A* entire gene allowed the identification of two deep-intronic variants (c.6806-810 A > G and c.6050-8058G > C). Although segregation analysis could not be performed, no additional variants that could explain the phenotype of this family were identified in other *loci*. Therefore, additional studies are needed to ascertain the clinical significance of these variants.

Screening intronic sequences also enables the proper detection of CNVs since it allows the determination of structural variants breakpoints at the nucleotide level, as well as the presence of inversions^35,36^. Recent studies have shown that CNVs in genes such as *EYS*^37,38^ or *USH2A*^28,39^, are indeed a significant event on the appearance of IRDs^27,40,41^. This is in agreement with our results showing the identification and accurate detection of their breakpoints of two novel, likely pathogenic, CNVs in *USH2A*. Therefore, it is highly recommended that the data analysis pipeline includes a suitable algorithm for the detection of these complex alleles.

Remarkably, of the 25 patients with previously detected monoallelic variants, 13 carried a second mutated allele in the same gene (*USH2A*). The majority of the unsolved patients carried a previously identified variant in *ABCA4*. This fact can be explained by the polymorphic nature of certain genes, and specifically of certain disease-causing reported variants. In the past, when the available genetic testing techniques were based on sequencing or genotyping a few exons per sample, detecting a sequence variant was a challenging task and it was interpreted as a causal mutation as long as it correlated with family segregation analysis and it was absent in 100 control individuals. To date, the proliferation of exome and genome sequencing projects and their use in the clinical setting have allowed unmasking some of the variants previously described as pathogenic and now considered as benign changes or at least variants of unknown significance (VUS) in certain populations^42,43^. In this regard, two variants in *ABCA4*, p.Ile156Val and p.Val2050Leu, have been traditionally considered disease-causing mutations^44,45^. However, an extensive revision of the literature^46–48^, the relatively high MAF according to 1000G (MAF = 0.019 in IBS and 0.029 in PUR respectively) and the fact that both mutations have been reported in *cis* with protein-truncating variants in STGD patients^46,49^, suggest that the clinical significance of these missense changes must be interpreted with caution especially in the context of genetic and reproductive counselling. Another explanation for the lack of success in detecting a second mutant allele in *ABCA4* may be that, even if these changes were certainly pathogenic, they may not be the cause of disease in our patients. This is especially relevant in those cases where genotype does not correlate with the phenotype, for example, for STGD associated mutations in RP patients. In this regard, the frequency of IRD carriers in the general population is known to be relatively high^50^, as demonstrated by the increased prevalence of IRDs in consanguineous communities^51,52^. Of note, we have detected two patients with the *ABCA4* variants p.Ile156Val and p.Val2050Leu, respectively, and additional mutations in other *loci*.

The identification of p.Ile156Val in family B can be considered a chance finding. The affected member of family B (II:1) harboured the recurrent single base pair deletion in *CNGB3*, c.1148del; p.Thr383Ilefs*13. This mutation is the most common variant underlying achromatopsia (ACH) worldwide^53,54^, accounting for over 70% of all *CNGB3* changes and about 40% of all ACH associated alleles^55^. Additionally, this variant has also been found in patients with juvenile macular degeneration^56^, macular malfunction^56^ and, recently, cone dystrophy (COD)^57^. Ophthalmologic examination of patient II:1 of family B confirmed the clinical diagnosis of COD. Although the pathogenicity of *CNGB3* c.1148del (p.Thr383Ilefs*13) seems convincing due to the large amount of supporting studies, two homozygous individuals have been detected in healthy control databases, one in ExAC and another one in EVS (entry 8:87,656,008AG/A), but the presence of pathogenic variants in healthy individuals has already been widely documented^43,58^.

Likewise, the contribution to the phenotype of the *ABCA4* p.Val2050Leu variant in the index patient of family C (II:1) is not entirely clear. This patient carries also a heterozygous variant in gene *FSCN2*. Nevertheless the pathogenicity of the *FSCN2* mutation could not be ascertained due to the lack of family members for segregation studies and the poor results of the *in silico* predictors (Table 1). Moreover, the association of this gene with IRDs is controversial^59,60^. Therefore, additional studies will be required to diagnose this simplex patient.

Interestingly, two families (F and Q) which were previously assessed with a customized panel are now genetically explained with a second mutation in *USH2A* (Table 2). The second hits consisted of both intronic variants (c.2167 + 5G > A; r.(spl?) and (c.12067-2 A > G; r.spl, respectively. Both variants should have been detected by the previous panel approach, but likely, they were bioinformatically filtered out due to the presence of duplicates.

In our cohort, the *USH2A* variant p.Cys759Phe was the most commonly mutated allele. Accordingly with other studies performed worldwide, this mutation is one of the most prevalent *USH2A* variants associated, in almost all of the cases, with non-syndromic RP^61,62^. Additionally, p.Cys759Phe variant has been frequently detected in compound-heterozygous state accompanied by a deleterious allele, while homozygous cases are rare and have been the subject of controversy^42^. In order to assess the hypothesis that the p.Cys759Phe variant is not pathogenic *per se* but it would be acting in *cis* with another non-coding pathogenic *USH2A* variant nearby, we analyzed in detail the deep intronic regions of this gene in solved cases harbouring this mutation. However, we were unable to identify any shared variant that met the criteria to be classified as pathogenic^19^. Therefore, if additional genetic load is acting together with the p.Cys759Phe, it is possibly that it is located in other regulatory regions.

Among the other *USH2A* mutations, two of them (p.Glu767Serfs*21 and p.Arg303Ser) have been detected in both syndromic and non-syndromic RP patients (Families M, N, O and P). The expression of the phenotype varies depending on the nature of the second mutation. Therefore, the greater the impact on the protein function, the greater the likelihood of developing the most severe condition, in this case, USH. Remarkably, the affected members of family P were compound heterozygous for one CNV (duplication of exons 46–47) and one missense (p.Arg303Ser), previously reported to be causative of USHII^61,63^. The index patient (family P-II:9) presented, besides typical arRP, bilateral sensorineural hearing loss. These findings were consistent with a diagnosis of USHII. However, the other two affected siblings (II:1 and II:7) were diagnosed of non-syndromic RP. The fact that a specific combination of mutations may be associated with a wide spectrum of symptoms in the same family, can be explained by the modulating effects of other genes and/or environmental factors on phenotypic expression^64^.

The main aim of this study was to evaluate the contribution of deep-intronic variants in a cohort with a previously detected heterozygous mutation in *ABCA4*, *USH2A* and *CEP290*. In this regard, only two predicted pathogenic mutations might be considered to be disease-causing in one patient (Family S, Supplementary Fig. S1). Prediction reports of intronic variants must be interpreted with caution in a clinical context and functional studies are mandatory. Moreover, although a study involving a larger number of samples would help to clarify the role of deep-intronic variants in the aetiopathogenesis of IRDs, the results presented here seem to indicate that deep-intronic variants have a small contribution in this group of patients.

In summary, the possibility of sequencing a number of entire genes represented an intermediate strategy between targeted sequencing and whole-genome sequencing. However, due to the high prevalence of carriers of mutations in IRD genes in the general population, the large amount of data generated with this panel, and the results obtained in this study, sequencing entire genes do not seem to be the approach of choice for detecting the second hit in IRD patients with monoallelic variants.

## Methods

### Subjects and clinical evaluation

A total of 29 unrelated Spanish families with different IRDs were involved in this study, including all available family members for segregation analysis. This cohort was composed of 25 index patients with one previous known mutation in *ABCA4*, *CEP290* or *USH2A* genes, and 4 IRDs patients that had not been studied before but with clinical suspicion of harbouring mutations in the genes included in the panel (Table 2). Prior to the study, written informed consents were obtained from all participants or their legal guardians. Study protocols followed the tenets of the Declaration of Helsinki and they were approved by the Institutional Review Boards of the University Hospital Virgen del Rocío (Seville, Spain).

Clinical diagnosis of retinal dystrophy was based on fundus examination, visual acuity, computerized testing of central and peripheral visual fields and electroretinography (ERG) findings. Furthermore, certain non-ocular features associated with retinal degenerations were evaluated in syndromic cases. Peripheral blood was collected from all subjects to extract genomic DNA using standard protocols. Previous analyses of the 25 subjects with known mutations were made by Asper Biotech Genotyping microarrays, Sanger sequencing of exon 13 of *USH2A* or by applying an earlier version of our custom panel^9,30^ (Table 2).

### Custom panel development

Our IRDs custom panel was designed using the SeqCap EZ application of the NimbleDesign software (Roche, NimbleGen, Madison, WI, USA). The intended covered sequences comprised three whole genes (*ABCA4*, *CEP290* and *USH2A*) known to have deep-intronic mutations associated with IRDs, as well as the coding exons and their adjacent 25 bp of 76 IRD genes (Supplementary Table S2). The genes were selected as previously described^9^; only those genes with pathogenic mutations in Spanish population were included. Besides, two known point mutations in deep-intronic regions of *OFD1* (c.935 + 706 A > G) and *PRPF31* (c.1374 + 654 C > G) were covered as well. A total of 1,239 regions were targeted, with a final panel size of 1,412,505 bp.

### Library preparation and sequencing

DNA library was performed according to the manufacturer’s protocol (NimbleGen SeqCap EZ Library SR version 5.1, Roche). Briefly, 1 μg of genomic DNA was sheared using Covaris S220 (Covaris, Woburn, MA, USA) to obtain an average fragment size of 180–220 bp. A multiplex DNA library pool, generated by mixing identical amount of DNA from several samples, was captured. Quantification of libraries was made using Agilent 2100 Bionalyzer (Agilent Technologies, CA, USA), qPCR and fluorimetric techniques. Sequencing was performed on the Illumina’s MiSeq or NextSeq instruments (Illumina, San Diego, CA, USA) using a MiSeq v2 (300 cycles) and NextSeq Mid-output v2 (300 cycles) reagent kits.

### Bioinformatic analysis

Data analysis was performed using our validated pipeline^30^ with some modifications. Burrows-Wheeler Aligner (BWA, version 0.7.12) was used to map sample reads against the hg19 human reference genome. BEDtools package (version 2.17.0) was used to analyze the percentage of reads on-target and the mean coverage in each sample. Duplicate reads were filtered out by employing PICARD’s MarkDuplicates command (version 1.95). Variant calling and filtering were carried out using GATK software (version 3.3.0) and reads with coverage <20X and strand bias (FS > 60.0) were discarded. SNVs and indels variants were then annotated using wANNOVAR^65^, and only those with MAF < 0.015 in 1000G, Exome Variant Server (EVS), Exome Aggregation Consortium (ExAC), genome Aggregation Database (GnomAD) and dbSNP remained for further analysis. The frequency of all candidate variants was also checked in the Collaborative Spanish Variant Server (http://csvs.babelomics.org/) including a local population database that contains population frequency information from the whole exomes of 267 unrelated individuals, representative of the healthy Spanish population (Medical Genome Project, MGP)^66^.

Copy-Number Variations (CNVs) were identified employing the coverage command of BEDtools. In this method, the number of reads for each chromosomal interval of the bed file was normalized using the average number of reads generated per sample. These data were then compared with the corresponding data of the other samples in the same sequencing run. A ratio around 1 implied normal dosage; deletions and duplications ratios were set on <0.6 and >1.40 respectively. All CNVs were checked in Database of Genomic Variants (DGV) and DECIPHER^67^.

### Variants prioritization and pathogenicity assesment

Prioritization was made with a step-by-step in-house pipeline. All variants of each sample were filtered by a Minor Allele Frequency (MAF) consistent with their disease (for IRD, MAF < 0.015). In patients with one known pathogenic variant in the coding sequence of *ABCA4*, *CEP290* or *USH2A*, variants in these genes were prioritized. Heterozygous exonic, splicing and intronic variants were selected for further analysis, as well as variants with low coverage (<20X). For intronic and synonymous variants, three online tools were used to assess splicing changes: NNSPLICE (http://www.fruitfly.org/seq_tools/splice.html) and two algorithms included in Human Splicing Finder (HSF and MaxEntScan; http://www.umd.be/HSF). Specific thresholds were defined based on a known deep-intronic variants validation study for two tools^19^: a minimum score of 2 and a score variation >15% for MaxEnt and a minimum score of 70 and score variation >10% for HSF, was necessary to pass the quality threshold. For NNSPLICE, we use default settings (cut-off >0.4) and a score difference between wild-type and mutated sequence >10% was needed to be considered for further analysis. The pathogenicity of novel candidate variants was predicted using Polyphen-2 (http://genetics.bwh.harvard.edu/pph2/), SIFT (http://sift.bii.a-star.edu.sg) and MutationTaster (www.mutationtaster.org/). Clinical significance of known variants was also assessed using ClinVar (https://www.ncbi.nlm.nih.gov/clinvar/) and/or Leiden Open Variation Database, LOVD (https://databases.lovd.nl/). Candidate variants were segregated in all available relatives by Sanger sequencing according to the manufacturer’s protocols (3730 DNA Analyzer, Applied Biosystems, Foster City, CA, USA).

To be considered causal variants, they must (i) segregate with the disease, (ii) be described as pathogenic or likely pathogenic in databases (ClinVar, OMIM) or be a novel mutation, or (iii) have clinical manifestations consistent with the ones described for this variant. Large deletions and duplications were inspected with Integrative Genomics Viewer (IGV). If no candidate variants were found in these three entire genes, mutations in other loci were taken into account as described above. The nomenclature of variants was adjusted to the Human Genome Variation Society (http://varnomen.hgvs.org/) guidelines using Mutalyzer (https://mutalyzer.nl/).

## Electronic supplementary material

Supplementary Information
