# Supplementary material for: Searching the second hit in patients with inherited retinal dystrophies and monoallelic variants in ABCA4, USH2A and CEP290 by whole-gene targeted sequencing
Source: Sci Rep. 2018 Sep 6;8:13312. doi: 10.1038/s41598-018-31511-5 (PMC6127285; doi:10.1038/s41598-018-31511-5)
Supplement: Supplementary file 1 — Supplementary Information [file 41598_2018_31511_MOESM1_ESM.pdf]

SUPPLEMENTARY INFORMATION

Searching the second hit in patients with inherited retinal dystrophies and monoallelic variants in *ABCA4*, *USH2A* and *CEP290* by whole-gene targeted sequencing

María González-del Pozo[a,b,#], Marta Martín-Sánchez[a,#], Nereida Bravo-Gil[a,b], Cristina Méndez-Vidal[a,b], Ángel Chimenea[a], Enrique Rodríguez-de la Rúa[c,d], Salud Borrego[a,b] and Guillermo Antiñolo[a,b*]

[a]Department of Maternofetal Medicine, Genetics and Reproduction, Institute of Biomedicine of Seville, University Hospital Virgen del Rocío/CSIC/University of Seville, Seville, Spain.
[b]Centro de Investigación Biomédica en Red de Enfermedades Raras (CIBERER), Seville, Spain.
[c]Department of Ophthalmology, University Hospital Virgen Macarena, Seville, Spain.
[d]Retics Patologia Ocular. OFTARED. Instituto de Salud Carlos III. Spain.

[#]These authors contributed equally to this work.

[*]**Corresponding author**:
Guillermo Antiñolo, MD, PhD
Department of Maternofetal Medicine, Genetics and Reproduction
University Hospital Virgen del Rocío
Avenida Manuel Siurot s/n
41013, Seville. Spain
E-mail: guillermo.antinolo.sspa@juntadeandalucia.es;
Phone: + 34 955 012 772

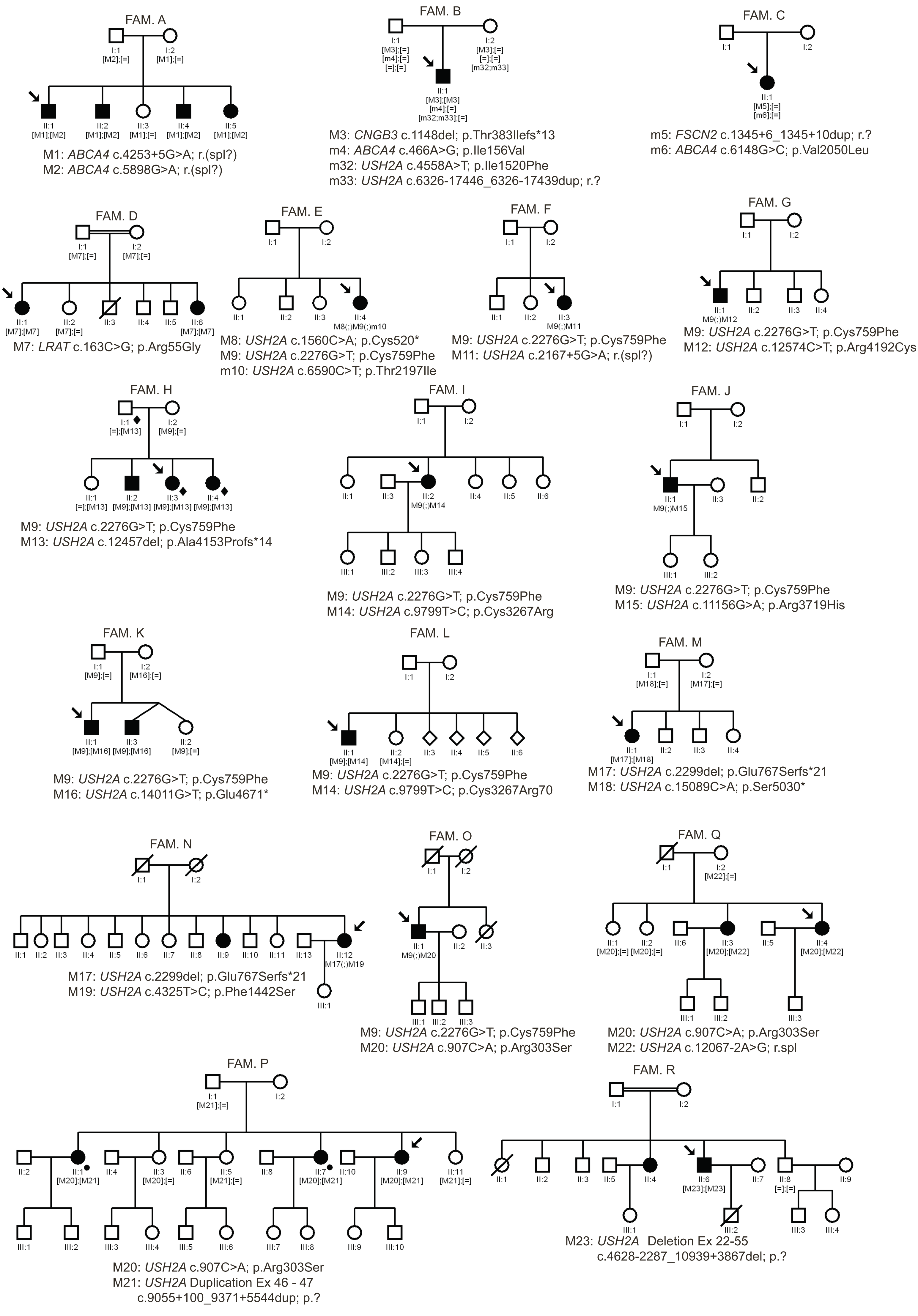
FAM. A
M1: ABCA4 c.4253+5G>A; r.(spl?)
M2: ABCA4 c.5898G>A; r.(spl?)
FAM. B
M3: CNGB3 c.1148del; p.Thr383Ilefs*13
m4: ABCA4 c.466A>G; p.Ile156Val
m32: USH2A c.4558A>T; p.Ile1520Phe
m33: USH2A c.6326-17446_6326-17439dup; r.?
FAM. C
m5: FSCN2 c.1345+6_1345+10dup; r.?
m6: ABCA4 c.6148G>C; p.Val2050Leu
FAM. D
M7: LRAT c.163C>G; p.Arg55Gly
FAM. E
M8: USH2A c.1560C>A; p.Cys520*
M9: USH2A c.2276G>T; p.Cys759Phe
m10: USH2A c.6590C>T; p.Thr2197Ile
FAM. F
M9: USH2A c.2276G>T; p.Cys759Phe
M11: USH2A c.2167+5G>A; r.(spl?)
FAM. G
M9: USH2A c.2276G>T; p.Cys759Phe
M12: USH2A c.12574C>T; p.Arg4192Cys
FAM. H
M9: USH2A c.2276G>T; p.Cys759Phe
M13: USH2A c.12457del; p.Ala4153Profs*14
FAM. I
M9: USH2A c.2276G>T; p.Cys759Phe
M14: USH2A c.9799T>C; p.Cys3267Arg
FAM. J
M9: USH2A c.2276G>T; p.Cys759Phe
M15: USH2A c.11156G>A; p.Arg3719His
FAM. K
M9: USH2A c.2276G>T; p.Cys759Phe
M16: USH2A c.14011G>T; p.Glu4671*
FAM. L
M9: USH2A c.2276G>T; p.Cys759Phe
M14: USH2A c.9799T>C; p.Cys3267Arg70
FAM. M
M17: USH2A c.2299del; p.Glu767Serfs*21
M18: USH2A c.15089C>A; p.Ser5030*
FAM. N
M17: USH2A c.2299del; p.Glu767Serfs*21
M19: USH2A c.4325T>C; p.Phe1442Ser
FAM. O
M9: USH2A c.2276G>T; p.Cys759Phe
M20: USH2A c.907C>A; p.Arg303Ser
FAM. Q
M20: USH2A c.907C>A; p.Arg303Ser
M22: USH2A c.12067-2A>G; r.spl
FAM. P
M20: USH2A c.907C>A; p.Arg303Ser
M21: USH2A Duplication Ex 46 - 47
c.9055+100_9371+5544dup; p.?
FAM. R
M23: USH2A Deletion Ex 22-55
c.4628-2287_10939+3867del; p.?

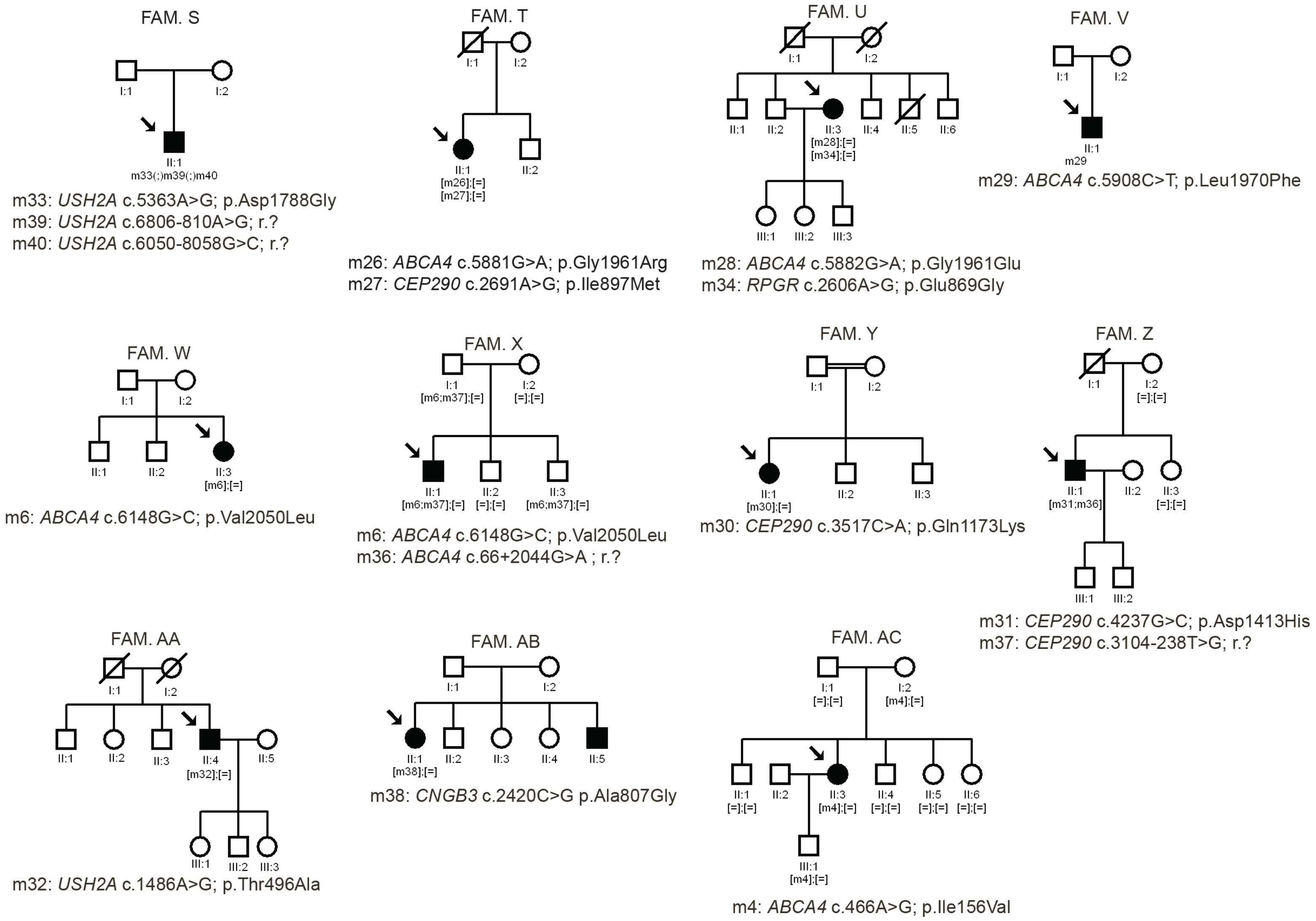

Supplementary Figure S1. Pedigrees of the 29 families included in the study. Variants coded by a upper case 'M' are considered likely causal whereas variants coded by a lower case 'm' have no demonstrated association with the disease in these patients. Symbols have been used to indicate intra-familiar variability: diamond symbols in family H refers to individuals with hearing loss; individuals in family P with arRP are indicated with a small black circle symbol, whereas the index patient have Usher syndrome.

Supplementary Table S1: Uncovered regions in *USH2A* , *ABCA4* and *CEP290* . The 41 studied regions (38 in *USH2A* , one in *ABCA4* and two in *CEP290)* lay within introns. Percentages of uncover introns are shown below.

***USH2A* (NM_206933)**	**Region (bp)**	**Localization**	**Intron lenght (bp)**	**uncovered intron (%)**	**uncovered intron (total %)**
chr1:215875496-215876307	811	intron 61-62	47653	1,7	1,86
chr1:215877262-215877335	73	intron 61-62	47653	0,15	
chr1:215926381-215926903	522	intron 58-59	15259	3,42	3,42
chr1:215976558-215978259	1701	intron 49-50	14610	11,64	12,25
chr1:215985250-215985338	88	intron 49-50	14610	0,6	
chr1:216014671-216014996	325	intron 46-47	6190	5,25	7,22
chr1:216015690-216015812	122	intron 46-47	6190	1,97	
chr1:216024163-216024662	499	intron 44-45	20973	2,38	4,67
chr1:216025381-216025512	131	intron 44-45	20973	0,62	
chr1:216026737-216026963	226	intron 44-45	20973	1,08	
chr1:216029429-216029552	123	intron 44-45	20973	0,59	
chr1:216084979-216085170	191	intron 38-39	33710	0,57	0,81
chr1:216089199-216089281	82	intron 38-39	33710	0,24	
chr1:216124625-216126490	1865	intron 37-38	30521	6,11	6,11
chr1:216151086-216151177	91	intron 35-36	22243	0,41	0,41
chr1:216181754-216182540	786	intron 32-33	45868	1,71	5,23
chr1:216185233-216185313	80	intron 32-33	45868	0,17	
chr1:216185339-216185595	256	intron 32-33	45868	0,56	
chr1:216185884-216186886	1002	intron 32-33	45868	2,18	
chr1:216189165-216189429	264	intron 32-33	45868	0,58	
chr1:216203588-216203597	9	intron 32-33	45868	0,02	
chr1:216277723-216278182	459	intron 21-22	78038	0,59	6,56
chr1:216303681-216303997	316	intron 21-22	78038	0,4	
chr1:216319183-216319755	572	intron 21-22	78038	0,73	
chr1:216320424-216322533	2109	intron 21-22	78038	2,7	
chr1:216323328-216324400	1072	intron 21-22	78038	1,37	
chr1:216325472-216325809	337	intron 21-22	78038	0,43	
chr1:216330496-216330753	257	intron 21-22	78038	0,33	
chr1:216458207-216458271	64	intron 11-12	38181	0,17	0,41
chr1:216459028-216459102	74	intron 11-12	38181	0,19	
chr1:216459727-216459744	17	intron 11-12	38181	0,04	
chr1:216478193-216478484	291	intron 9-10	29512	0,99	1,43
chr1:216478607-216478737	130	intron 9-10	29512	0,44	
chr1:216513169-216513199	30	intron 4-5	37298	0,08	5,4
chr1:216513371-216515356	1985	intron 4-5	37298	5,32	
chr1:216543674-216543722	48	intron 3-4	53428	0,09	0,24
chr1:216559922-216559977	55	intron 3-4	53428	0,1	
chr1:216578264-216578289	25	intron 3-4	53428	0,05	
**ABCA4 (NM_000350)**	**Region (bp)**	**Localization**	**Intron lenght (bp)**	**uncovered intron (%)**	**uncovered intron (total %)**
chr1:94540023-94540628	605	intron 11-12	14372	4,21	4,21
**CEP290 (NM_025114)**	**Region (bp)**	**Localization**	**Intron lenght (bp)**	**uncovered intron (%)**	**uncovered intron (total %)**
chr12:88492109-88492138	29	intron 26-27	5838	0,5	0,5
chr12:88526846-88527217	371	intron 6-7	5424	6,84	6,84

Supplementary Table S2: Genomic regions of each gene included in the targeted-panel design. In *ABCA4*, *CEP290* and *USH2A*, chromosome coordinates are given, as they are included as entire genes. Regarding the 76 remaining genes, exons coded by a specific transcript (NM reference sequence) were contained in the final design. Additionally, two deep-intronic mutations were also added: *OFD1* (c.935+706A>G) and *PRPF31* (c.1374+654C>G). * Genes not included in previous panels.

Gen	Genomic Regions
ABCA4	chr1:94,458,393-94,586,688
ABHD12	NM_015600
*ACBD5**	NM_145698
AIPL1	NM_014336
ARL6	NM_177976
BBS1	NM_024649
BBS10	NM_024685
BBS12	NM_152618
BBS2	NM_031885
BEST1	NM_001139443
*C1QTNF5**	NM_015645
C2ORF71	NM_001029883
CA4	NM_000717
CDH23/USH1D	NM_022124
*CDHR1**	NM_033100
CEP290	chr12:88,442,793-88,535,993
CERKL	NM_201548
CHM	NM_000390
CLRN1	NM_001195794
CNGA1	NM_001142564
*CNGB1**	NM_001297
CNGB3	NM_019098
CRB1	NM_201253
CRX	NM_000554
DFNB31	NM_015404
EYS	NM_001142800
FSCN2	NM_001077182
GPR98	NM_032119
GUCA1A	NM_000409
*GUCA1B**	NM_002098
GUCY2D	NM_000180
IMPDH1	NM_000883
LCA5	NM_001122769
LRAT	NM_004744
MERTK	NM_006343
MKKS	NM_170784
MYO7A	NM_000260

NMNAT1	NM_022787
NR2E3	NM_016346
NRL	NM_006177
OFD1	NM_003611
PCDH15/USH1F	NM_001142763
*PDE6A**	NM_000440
PDE6B	NM_000283
*PDZD7**	NM_001195263
*PEX6**	NM_000287
*PRCD**	NM_001077620
PROM1	NM_006017
PRPF3	NM_004698
PRPF31	NM_015629
PRPF8	NM_006445
PRPH2	NM_000322
*PRPS1**	NM_002764
*RAB28**	NM_001017979
RBP3	NM_002900
RD3	NM_001164688
RDH12	NM_152443
RGR	NM_002921
RHO	NM_000539
RLBP1	NM_000326
ROM1	NM_000327
RP1	NM_006269
RP2	NM_006915
RP9	NM_203288
RPE65	NM_000329
RPGR	NM_001034853
RPGRIP1	NM_020366
RS1	NM_000330
SAG	NM_000541
SAMD11	NM_152486
SNRNP200	NM_014014
SYNE2	NM_182914
TOPORS	NM_005802
TULP1	NM_003322
USH1C/DFNB18/harmonin	NM_153676
USH1G/SANS	NM_173477
USH2A	chr1:215,796,236-216,596,738
*VCAN**	NM_004385
ZNF408	NM_001184751
